# German version of the whiplash disability questionnaire: reproducibility and responsiveness

**DOI:** 10.1186/1477-7525-11-36

**Published:** 2013-03-08

**Authors:** Michael McCaskey, Thierry Ettlin, Corina Schuster

**Affiliations:** 1 Research Department Reha Rheinfelden, Salinenstrasse 98, Rheinfelden 4310, Switzerland; 2 Institute for Human Movement Sciences, Department of Health Sciences and Technology, ETH Zurich, Zurich, Switzerland; 3 Department of Behavioural Neurology, Medical Faculty, University of Basel, Basel, Switzerland; 4 Institute for Rehabilitation and Performance Technology, Department of Engineering and Information Technology, Bern University of Applied Sciences, Bern, Switzerland

**Keywords:** Whiplash, Assessment, Questionnaires, Responsiveness, Reliability, Kraniozervikales Beschleunigungstrauma, Assessments, Fragebogen, Reliabilität, Veränderungssensitivität, Reproduzierbarkeit

## Abstract

**Background:**

The Whiplash Disability Questionnaire (WDQ) poses a validated tool for the assessment of patients who experience whiplash-associated disorders. A German translation and cross-cultural adaptation was recently produced and presented high validity and internal consistency. As a follow-up, the presented study tests the translated Whiplash Disability Questionnaire’s (WDQ-G) retest reliability and responsiveness to change.

**Methods:**

The WDQ-G was assessed on three different measurement events: first upon entry (ME1), second four days after entry (ME2), and third at discharge (ME3). Test-retest reliability data from ME1 and ME2 was analysed in a group of stable patients to obtain the intraclass correlation coefficient (ICC) and the standard error of measurement (SEM). To test the instrument’s responsiveness, WDQ-G change data were compared to concurrent instruments. The probability of each instrument, to correctly distinguish patients of the stable phase (ME1 to ME2) from patients who deemed to have improved between from ME1 to ME3, was analysed.

**Results:**

In total, 53 patients (35 females, age = 45 ± 12.2) were recruited. WDQ-G scores changed from ME1 to ME2 by 5.41 ± 11.6 points in a stable group. This corresponds to a test-retest reliability of ICC = 0.91 (95% CI = 0.80–0.95) with a SEM of 6.14 points. Minimal Detectable Change, at 95% confidence, was calculated to be 17 points change in scores. Area under Receiver Operator Characteristics of the WDQ-G’s responsiveness revealed a probability of 84.6% (95% CI = 76.2%–93%) to correctly distinguish between improved and stable patients. Optimal sensitivity (73.2%) and specificity (76.2%) was established at 11-point change.

**Conclusions:**

High retest reliability and good responsiveness of the WDQ-G support clinical implementation of the translated version. The data suggest, that change in total score greater than eleven points can be interpreted as clinical relevant from a patient’s perspective. Minimal Important Change is suggested at 15 points where there is still high specificity and a 90% confidence MDC.

## Abstract (de)

**German abstract:**

**Hintergrund**

Der Whiplash Disability Questionnaire (WDQ) stellt einen validierten Fragebogen zur Erfassung der Alltagseinschränkungen bei Patienten nach kraniozervikalem Beschleunigungstrauma (KZBT) dar. Eine deutsche Übersetzung und kulturelle Anpassung mit hoher Validität und interner Konsistenz wurde bereits erstellt und getestet. In dieser Anschlussstudie soll die übersetzte Version auf Testwiederholung und Veränderungssensitivität überprüft werden.

**Methoden**

Die deutsche Version des WDQ (WDQ-G) wurde an drei verschiedenen Messzeitpunkten getestet: zuerst nach Eintritt (MZP1), vier Tage nach Eintritt (MZP2) und beim Austritt (MZP3). Für die Testwiederholung (Test-Retest) wurde die Veränderung der Punktzahl von MZP1 bis MZP2 in einer Gruppe stabiler Patienten untersucht und der Intraklassenkorrelationskoeffizient (ICC) sowie der Standardfehler der Messungen (SEM) berechnet. Für die Veränderungssensitivität wurde die Veränderung der Punktzahl des WDQ-G mit dem von konkurrierenden Fragebogen verglichen. Dabei wurde die Fähigkeit der Fragebogen untersucht, die stabilen Patienten aus der ICC-Analyse von den Patienten zu unterscheiden, die gemäss eigenen Angaben sich nach der Behandlung besser fühlten.

**Resultate**

Insgesamt wurden 53 Patienten (35 weiblich, Alter = 45 ± 12.2) rekrutiert. Die WDQ-G Punktezahl veränderte sich von MZP1 bis MZP2 um 5.41 ± 11.6 in der stabilen Gruppe. Dies entspricht einem ICC von 0.91 (95% CI = 0.80–0.95) mit einem SEM von 6.14 Punkten. Für eine statistisch relevante Veränderung (MDC) waren 17 Punkte nötig (95% Konfidenz). Die Wahrscheinlichkeit des WDQ-G, die Patienten korrekt zu unterscheiden, lag bei 84.6% (95% CI = 76.2%–93.0%). Optimale Sensitivität (73.2%) und Spezifizität (76.2%) sind bei einer Veränderung von 11 Punkten zu erreichen.

**Schlussfolgerung**

Hohe Reliabilität und gute Veränderungssensitivität unterstützen die Empfehlung, den WDQ-G für die Einschätzung von KZBT Patienten zu nutzen. Die Resultate suggerieren eine Veränderung von 11 Punkten als relevant aus Patientenperspektive. Um systematische Fehler des Fragebogens zu berücksichtigen, sollte eine Veränderung unter 15 Punkten (90% Konfidenz) nicht als relevant interpretiert werden.

## Background

The Swiss National Accident Insurance Fund registered roughly 11,000 new cases of whiplash-associated disorders (WAD) causing costs of up to 270 million Swiss Francs [1] every year. Despite a gradual increase of these numbers over the last three decades, understanding of the condition still remains poor. One of the few consensus reached on the handling of WAD is that diagnostic procedures and imaging techniques do not produce valid outcomes needed for adequate diagnosis and planning of treatments [2,3]. WAD, defined as consequences of whiplash-like accidents, often cause multiple limitations on various domains of life including function, activity, and participation [2]. It is important for any clinician to properly monitor a patient’s development, be it improvement or increase of symptoms. Pain alone as an outcome does not provide sufficient specificity, or sensitivity and therefore lacks prognostic value [4,5]. A more global measure is required that also accounts for interference with daily living [2]. In the past two decades there have been two noteworthy projects attempting to produce recommendations concerning handling cases of WAD: the Quebec Task Force (QTF) on WAD [3] and, as part of the Bone and Joint Decade 2000–2010, the Task Force on Neck Pain and Its Associated Disorders [2]. Both publications contributed vastly to the understanding of the complex nature of whiplash and the classification of subgroups in patients allowing a more specific therapy. However, the QTF does not provide instrument with respect to the predictive value, sensitivity, specificity, and acceptability of diagnostic tests [2]. Accordingly, clinical decision-making still lacked an appropriate monitoring tool sensitive to change regarding self-perceived health status. Based on Hoving’s qualitative research [6], Pinfold et al. proposed the Whiplash Disability Questionnaire (WDQ) “designed to evaluate whiplash-related disability” [7]. The WDQ is a self-administered outcome measure to evaluate pain intensity and limitations due to a WAD in different domains: current pain level, personal care, role performance, mobility, sleep disturbances, tiredness, social and leisure (sporting and non-sporting) activity, emotional and cognitive impairments. It is a self-administered disease-specific questionnaire consisting of 13 items to which the patients respond by circling their personal agreement on an 11-point scale (zero to ten) for each item. The higher the total score the higher the subjective perceived impairment. Its psychometric qualities have been evaluated and it has been approved to be a valid tool to describe and monitor the perceived participation in everyday activity of patients with WAD [7,8].

Considering these positive results of the English version and after identifying the lack of an assessment with comparable quality criteria for German speaking regions, a project was initiated in 2004 to produce a culturally adapted and evaluated German translation of the WDQ. In an article published by Schuster et al. [9]*(German translation, cross-cultural adaptation and validation of the Whiplash Disability Questionnaire)* a standardised six-step translation process is described to produce a German version of the Whiplash Disability Questionnaire, the WDQ-G. The translated questionnaire was tested on 70 patients with WAD. The report suggests good concurrent validity (r = 0.71–0.74), high internal consistency (α = 0.89), and recommends its application with German-speaking patients with WAD.

To allow cross-national comparison of outcomes and international collaboration in clinical research, the translations of individual assessments must produce reproducible questionnaires and reflect the content of the original ones [10]. The aims of the present study were to evaluate the test-retest reliability of the German version of the WDQ as an indicator for reproducibility in a stable study population. Further, its ability to recognise minimal clinical important change (MIC) after a rehabilitative intervention was evaluated with concurrent questionnaires.

## Methods

### Design

The patient study for evaluation of psychometric properties was conducted between June 2006 and September 2008 in a rehabilitation centre in the German-speaking part of Switzerland. Four consecutive measurement events (ME) were recorded (entry ME1, two to four days after entry ME2, at discharge ME3, and six month after discharge ME4). Here, only the first three MEs are presented for analysis of test-retest reliability and responsiveness.

### Participants

For the study, a sample of German speaking men and women after initial or repeated WAD, QTF II (neck complaints and musculoskeletal signs [3]), with or without mild traumatic brain injury – MTBI [11]) were included if they were older than 18 years and gave written informed consent. Patients with additional neurological conditions (cerebrovascular insult or brain tumours), systemic diseases (e.g. Fibromyalgia, Rheumatoid diseases), psychiatric comorbidities, or reduced attention capacities observed during the examination, were excluded from participation. Furthermore, patients were excluded if they required mobility aids (e.g. walking sticks, wheel chair, wheeled walkers).

The local ethics committee of the Canton Aargau approved the project (reference number 2005/039). All procedures were in accordance with the Declaration of Helsinki.

### Outcome measurements and measurement events

After given informed consent, patient characteristics and accident history was recorded on the case report form prior to ME1. The physician in charge of the entry-examination asked patients to fill in the questionnaires for ME1 data prior to the multidisciplinary rehabilitation program (active and passive physiotherapy and psychological treatment). The investigator of the study handed out the questionnaires for ME2 data two to four days after entry (ME2). At the end of the inpatient period (3 – 4 weeks after entry), participants were asked to fill in the third questionnaire set (ME3). At each ME, patients were asked to complete a set of four questionnaires: the WDQ-G, the North American Spine Societies Questionnaire (NASS, a cervical problem-specific questionnaire), the Medical Outcomes Study (MOS) 36-Item Short Form Health Survey (SF-36), and the Visual Analogue Scale (VAS) for pain. The NASS subscale for pain and disability (NASS-PF) was used as one of the concurrent instruments for responsiveness analysis. It consists of eleven items with item scores ranging from one (“I can perform without pain”) to six (“Due to my pain level I cannot perform at all.”). A high score indicates a high degree of impairment [10]. The official German translation has shown to be a reliable measure [12,13] and can be used for patients with WAD [14,15]. The bodily pain dimension of the SF-36 physical health component (SF-36BP) was the second comparator used for responsiveness analysis. It consists of two items (pain magnitude and pain interference) which score’s are coded, summed and transformed to a scale from 0 (worst possible health state measured by the item) to 100 (best possible health state). The SF-36 has shown to be a reliable and valid measure of disability in different languages for different pathologies, including WAD [16-23]. The non-validated health transition item (HTI) of the SF-36 was also included in the analysis to record the change in global health perception from a patient’s perspective. At ME1 and ME2, the item asked: “Compared to one ear ago, how would you rate your health in general now?”. At ME3, after treatment, the item addressed the change occurred since the beginning of the treatment: “Compared to before your rehabilitation therapy, how would you rate your health in general now?”. Five possible answers allowed the patients to report whether they felt their condition has strongly improved (=1), moderately improved (=2), remained unchanged (=3), deteriorated by some degree (=4), or even strongly deteriorated (=5).

On the VAS, the third comparator, patients reported actual subjective pain intensity indicated on a horizontal 10 cm straight line anchored by two extremes of pain: “no pain” (0 cm) and “pain as bad as it could be” (10 cm) [23,24].

Anonymised and completed SF-36 and NASS questionnaires were scanned to upload by secure data transfer to an independent company (RehabNET AG, Zürich, Switzerland) for data assembly and subsequently returned for in-house analysis. Questionnaires for demographic and descriptive statistics as well as VAS and WDQ data were recorded manually within the clinic using Microsoft Excel 2003.

### Data analysis

Patients were dichotomised into two groups based on their responses on the HTI. The improved group was assembled from patients who responded on the HTI with moderately or strongly improved after treatment (ME3). The stable group, on the other hand, was assembled from patients who presented no change in HTI score from ME1 to ME2. Returned questionnaires were classified as incomplete if more than 2 items were missing on the WDQ-G or any item was missing on the reference questionnaires (VAS, SF-36BP bodily pain, and NASS-PF pain & function). Only complete questionnaire sets were included for analysis. Change in scores from one ME to another was calculated and statistically compared with the paired Student *t*-test. All statistical analyses were computed on the IBM Statistical Package for Social Sciences version 20, 2011 (IBM Corp.©) with p ≤ 0.05.

### Reliability

The stable group (no change from ME1 to ME2) provided test-retest data to calculate the Intraclass Correlation Coefficient (ICC) as an indicator for reproducibility. The ICC_A,1_ two-way random model was applied, where A stands for absolute agreement [26]. Single measure values of 0.65 or above were regarded as statistically acceptable [21]. Confidence intervals (CI) were also calculated to provide upper and lower limits of the 95% certainty and the Standard Error of Measurement (SEM) was calculated as $SD_{\text{stable}}\times \sqrt{1-\text{IC}C_{\text{stable}}}$, where SD_stable_ represents the baseline score of the stable group [27].

### Responsiveness

As there exists considerable confusion regarding the nomenclature for reporting and quantifying responsiveness [27], it seems appropriate to introduce the herein applied terminology for this report. Recommendations of Crosby et al. [28] were used as guidance with additional consideration of the combined approach recommended by de Vet et al. [29] without claiming superiority over other available terminology [27,30].

In their review on change in health-related quality of life, Crosby et al. recommend two major approaches of responsiveness: Criterion-referenced change (or anchor-based methods) and precision-referenced change (or distribution-based methods). Criterion-referenced change includes cross-sectional and longitudinal approaches, comparing the instrument under investigation with concurrent instruments. Precision-referenced change describes estimates based on statistical significance of the instrument under investigation. Estimates based on the combined approach are termed as MIC, i.e. a criterion-referenced change greater than precision-referenced change could be presumed meaningful [28].

Precision-referenced change was analysed by calculating the Minimal Detectable Change (MDC) as $1.96\times \sqrt{2\times \text{SEM}}$, which is related to the retest ICC assessed in the stable group between ME1 and ME2 [29]. As an indicator for magnitude of change the Standardized Response Mean (SRM) of the non-dichotomised group was calculated as the ratio of observed change to ME3 and its SD. The SRM has the advantage over other effect size coefficients that it is independent of group size and proves especially valuable when compared to concurrent measures [27]. Between-difference variability of the individual SRM was compared using Student’s t-distribution for qualitative comparison of the instruments’ precision [27,31].

A small SRM would reflect high variability of the change scores. SRM higher than 0.5 was presumed to be adequate, SRM higher than 0.8 represents large responsiveness [32].

For the criterion-referenced change, the WDQ change scores were compared to the ones of the VAS, SF-36BP, and NASS-PF. First, Pearson’s correlation coefficients between change scores from ME1 to ME3 of the WDQ-G and VAS, SF-36BP, and NASS-PF were calculated [27] from the non-dichotomised group to evaluate whether the instruments respond in a similar way. To compare the instruments’ ability to distinguish between improved and stable patients, the Receiver Operating Characteristics (ROC) curve was plotted. Cut-off points for all instruments were determined as the point on the curve nearest to the upper left-hand corner where optimal sensitivity and specificity is expected (where sensitivity + (1-specificity) is minimal) [27]. A cut-off point greater than or equal to MDC was considered to be meaningful (=MIC) [28]. Comparison of the area under the ROC curve (AUC) was used to assess the WDQ-G’s responsiveness as compared to the traditional instruments, i.e. their probabilities to correctly distinguish between the two phases (stable from ME1 to ME2 and improved from ME1 to ME3) according to change in scores referenced to the HTI.

## Results

### Patient study

The screening period from 2006 to 2008 revealed 159 patients diagnosed with WAD. After selection for study criteria, the study cohort consisted of 70 patients who agreed to participate. No dropouts were recorded, but 17 patients failed to return complete data sets leaving a dataset of 53 patients for analysis (35 females, mean age 45 ± 12.2) referred to as non-dichotomised group. For the recruited patients, the time since accident ranged from 22 days to 18 years, mean time was 99 weeks for the non-dichotomised group. Mean amount of days between ME1 and ME2 was 3.42 (±2.1) days and 21.64 (±8.3) days from ME2 to ME3. At time of admission, patients’ average employability level was 37.65% (±37.4) with 7 patients still employed 100% (42 h/week) and 24 patients reporting not being able to work at all (0% employability). On the self-reported questionnaire, 10 patients (18%) indicated to have had MTBI from the accident and 7 patients reported still being involved in litigation.

After comparing MEs for dichotomisation, one improved group of N = 41 (from ME1 to ME3, 27 females) and one stable group of N = 42 (from ME1 to ME2, 31 females) patients were assembled for analysis.

### Test retest reliability

Table 1 summarises the results of ME1 to ME3 showing pre-treatment, retest, and post-treatment scores and score changes on all questionnaires. From ME1 to ME2 the mean change score for all participants was 6.06 points (±11.3, 95% CI = 2.95 to 9.16, p < 0.001) and 5.41 (±11.6, 95% CI = 1.793 to 9.11, p < 0.001) for stable patients. This corresponds to an ICC of 0.91 (95% CI = 0.80 to 0.95, p < 0.001) for the stable group and 0.92 (95% CI = 0.82 to 0.96, p < 0.001) for the non-dichotomised group. For the non-dichotomised group, maximum and minimum change from ME1 to ME2 was 47 and -16, from ME1 to ME3 it was 70 and 4, respectively. The WDQ-G SEM value for the non-dichotomised group was 6.82 and 6.16 for the stable group. Two outliers were identified with changes greater than 1.5 × the inter quartile range from ME1 to ME2 with 27 and 47 points change in scores.

**Table 1:** Summary of outcome scores from ME1 to ME3

**Instrument score**	**ME1 score, mean (SD)**	**ME2 score, mean (SD)**	**ME3 score, mean (SD)**	**ME1 to ME2, mean (SD)**	**ME1 to ME3, mean (SD)**
**WDQ-G**					
All	72.23 (22.3)	66.17 (24.2)	50.13 (29.1)	6.06 (11.3)*	22.09 (18.6)*
Improved^$^	69.62 (19.9)	63.31 (22.2)	43.40 (25.3)	6.32 (12.4)*	26.22 (18.2)*
Stable^£^	76.68 (20.1)	71.27 (22.6)	56.01 (28.9)	5.41 (11.6)*	20.67 (19.1)*
**SF-36BP**					
All	24.96 (13.6)	26.68 (16.0)	45.30 (21.9)	-1.72 (10.3)	-20.34 (20.4)*
Improved^$^	25.95 (11.9)	27.24 (13.4)	51.32 (18.4)	-1.29 (10.8)	-25.37 (19.5)*
Stable^£^	22.50 (11.5)	23.00 (13.1)	42.02 (21.9)	-.050 (10.8)	-19.52 (20.7)*
**NASS-PF**					
All	3.44 (0.8)	3.39 (0.8)	2.98 (1.0)	0.05 (0.4)	0.46 (0.6)*
Improved^$^	3.36 (0.8)	3.31 (0.8)	2.79 (0.9)	0.04 (0.4)	0.58 (0.6)*
Stable^£^	3.56 (0.8)	3.54 (0.8)	3.12 (1.0)	0.02 (0.4)	0.43 (0.6)*
**VAS**					
All	5.79 (1.9)	5.65 (2.4)	3.54 (2.7)	0.14 (1.6)	2.25 (2.5)*
Improved^$^	5.85 (1.7)	5.55 (2.3)	2.82 (2.0)	0.29 (1.7)	3.03 (2.2)*
Stable^£^	6.04 (1.8)	5.95 (2.3)	4.01 (2.7)	0.09 (1.7)	2.03 (2.6)*

*stat. significant on the level of 0.01; *ME* Measurement Event; *SD* Standard Deviation; *WDQ-G* Whiplash Disability Questionnaire; *SF-36BP* 36 Item Short Form Health Survey Bodily Pain Subscale; *NASS-PF* North American Spine Society Questionnaire Pain and Function Subscale ^$^HTI < 3 from ME1 to ME3; ^£^HTI > 3 from ME1 to ME2.

### Responsiveness

Pearson’s r for mean WDQ-G change of the non-dichotomised group from ME1 to ME3 with the mean change of the SF-36BP was 0.50 (p < 0.001), 0.69 (p < 0.001) for the NASS-PF, and 0.74 (p < 0.001) for the VAS. Change in score from ME1 to ME3 was significant for all measurement events with the highest t-value for the WDQ-G (t = 8.66) followed by the SF-36BP (t = 7.25), the VAS (t = 6.64) and the NASS-PF (t = 4.30). Comparison of the SRM suggests that the WDQ-G was the most responsive measure with a significantly greater SRM than the NASS-PF (95% CI = 0.22–0.65, p < 0.01) and the VAS (95% CI = 0.08–0.48, p < 0.01) but only insignificantly greater than the SF-36BP (95% CI = 0.12–0.42, p = 0.27). Responsiveness results for the investigated outcomes are summarized in Table 2.

**Table 2:** Responsiveness statistics

**Outcome**	**WDQ-G**	**SF-36BP**	**NASS-PF**	**VAS**
*Precision-referenced (non-dichotomised from ME1 to ME3)*				
SRM	1.19	1.00	0.75*	0.91*
*Precision-referenced (stable ME1 to ME2)*				
ICC (95% CI lower-upper)	0.91 (0.80–0.95)	0.76 (0.61–0.85)	0.94 (0.89–0.97)	0.79 (0.61–0.89)
SEM	6.14	5.58	0.19	0.82
MDC	17	16	0.53	2.26
*Criterion-referenced (improved from ME1 to ME3 vs. stable from ME1 to ME2)*				
ROC cut-off (MIC)	11 (17)^§^	16 (16)^§^	0.15 (0.6)^§^	1.850 (2.3)^§^
ROC AUC	0.85 (0.76–0.93)	0.880 (0.81–0.95)	0.78 (0.69–0.88)	0.87 (0.79–0.94)

*significant difference compared to WDQ-G SRM on the level of 0.01; *ME* Measurement Event; *SD* Standard Deviation; *WDQ-G* Whiplash Disability Questionnaire; *SF-36BP* 36 Item Short Form Health Survey Bodily Pain Subscale; *NASS-PF* North American Spine Society Questionnaire Pain and Function Subscale; *SRM* Standardised Response Mean; *ICC* Intraclass Correlation Coefficient; *SEM* Standard Error of Measurement; *MDC* Minimal Detectable Change; *ROC* Receiver Operating Characteristics; ^§^*MIC* Minimal Important Change (equals numbers in brackets); *AUC* Area under Curve of ROC.

**Figure 1:**
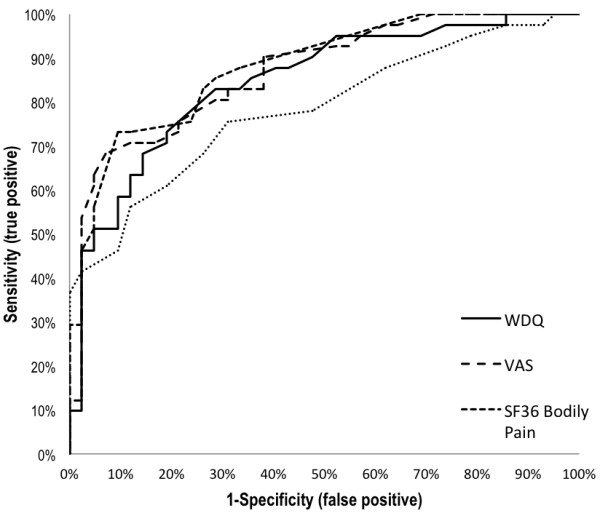
**Receiver Operating Characteristics curves comparison of change scores.** True positive value is “Improved”; True negative value is “Stable”.

ROC curves for the assessed instruments are presented in Figure 1. The figure shows that all instruments have similar high probabilities to correctly assess patients as improved with AUC values higher than 0.7, whereas the NASS-PF presented the lowest AUC. The determined cut-off value for the WDQ-G has a sensitivity of 0.73 and specificity of 0.76. Thus, 26.8% of the criterion-referenced improved patients have false-negative changes (observed change is lower), and 23.8% have false-positive changes (observed change is higher). By raising the cut-off to MDC (i.e. raised to MIC), the probability of false positives is reduced (11.9%) and the probability of false negatives is increased (38%) [33].

## Discussion

Analysis of the instrument’s systematic error revealed strong correlations between test and retest suggesting good reproducibility. Although the ICC calculated for the WDQ-G (ICC = 0.91) is slightly lower than the short-term ICC reported for the Australian version by Willis et al. [11] (ICC = 0.96), it is still acceptable for clinical measures [30].

Sample size was comparable to previous WDQ studies [6-8] and provides a representative German-speaking population with WAD. However, the considerably reduced sample size after exclusion of incomplete questionnaires may suggest reduced power of the reported outcomes. Indeed, to allow 95% certainty for the ICC to be greater than 0.86, a sample size of 73 patients would have been required. Still, with 42 patients included there is a 95% certainty for the ICC to be greater than 0.80, which could still be deemed sufficient [30]. This sample size is also justified in the light of Nunnally and Bernstein’s observations, that ICCs greater than 0.80 are less susceptible to measurement errors [34].

The inclusion criteria did not exclude patients with self-reported MTBI, although there is no clear definition as to whether this can be classified as QTF II and attentional deficits could be expected. However, all participating patients underwent medical examination as part of the admission process to the clinic and were deemed cognitively able to fill in all questionnaires [11].

The criterion-referenced ROC curve revealed that, in terms of sensitivity and specificity, the optimal cut-off point for the WDQ-G would be at 11 points change in scores. This is less than the precision-referenced MDC, which also accounts for systematic error. Consequently, the cut-off should be raised to MDC to reduce effects of the instruments measurement errors. Here we face the problem that if we want to consider variability for estimation of change and define MIC equal to MDC, the instrument’s ability to identify true improvement, as defined by subjective judgment of the patient, is reduced. The original version of the WDQ was assessed for responsiveness using the MDC for 90% CI with 1.64×SEM, thus suggesting a change of 15 points to be relevant [8]. Using the same constant for the German version yields similar results: 14.3 points. A MIC of 15 point in the present study corresponds to sensitivity of 64% and 86% specificity.

All instruments had large *t*-test values suggesting high responsiveness to the treatment for all groups from ME1 to ME3. Only the WDQ-G significantly changed in scoring within the stable groups from ME1 to ME2. This could be related to systematic score change, i.e. the high variability in change even in stable patients. As similar variability was reported for the original version [8], this might pose a potential weakness of the questionnaire to correctly identify improved patients with high sensitivity.

Although the results presented herein suggest good responsiveness of the WDQ-G, they are still not conclusive. Some of the aspects not addressed include baseline impairments, regression to the mean and direction of change. It should be investigated whether patients with more severe impairment require a greater change in score to be considered clinically meaningful improved than those with less severe pain. On the other hand, patients with greater impairments at baseline also have more opportunity to improve. Speer et al. suggest that baseline scores should therefore be adjusted for regression to the mean, e.g. by using the Edwards-Nunnally method [35]. It should also be analysed whether deterioration has different cut-off values than improvement [28]. Further, the use of self-reported global health state items as external criteria has been criticized extensively. Quite rightly so, as any anchor-based approach is only as good as the selected anchor [36]. One of its major flaws is the lack of accepted psychometric properties. Individual response might depend on current mood or a recent event that may have caused problems not related to WAD. This kind of bias cannot be excluded in a single item. In an attempt to improve this situation, we reflected on combining the external health transition item of the SF-36 with one of the validated and reliable instruments assessed alongside the WDQ-G. But applying, for instance, VAS change as external criteria would only address pain, whereas an improvement (or deterioration) in pain does not automatically mean reduced impairment due to WAD. From an individual perspective, the health transition item provides a good overall indication of the patient’s perspective on his or her health status [28] and has been applied in similar studies investigating responsiveness of health related quality of life measures [37,38].

Because criterion-referenced responsiveness is independent of time and intervention [27], it is possible to compare the group used for testing the instrument’s measurement precision (i.e. stable group) with a group containing some of the patients at a later point in time (i.e. after treatment as an improved group). Still, this method could be criticised as it rather compares the ability to distinguish between two phases (ME1 to ME2 versus ME1 to ME3) than between two groups after a specific intervention and pre-specified time frame. As the study’s aim was not to investigate the efficacy of a particular treatment, this method seemed appropriate to test the WDQ-G’s discriminatory performance.

A further limitation of the study design is the non-standardised intervention. Although rehabilitation programs are often similar across the clinics in a language specific region, they are seldom exactly the same. Thus, the studies findings are not necessarily applicable to all types of interventions. Included patients underwent a multidisciplinary therapy program consisting of active physiotherapy and exercises, passive treatments such as massage or thermal treatments, and psychological therapy. Husted et al. [27] point out that referencing the instrument under investigation to an external anchor reflects “a property of measure and has a meaning in a wider range of settings” than precision-referenced outcomes. This also justifies the application of more than just one statistics for responsiveness. Where the *t*-test is said to be a minimal indicator for responsiveness, this study also addressed the magnitude of change using the SRM. The SRM is advantageous over other effect size methods for its ability to reflect measurement precision (95% CI) and thereby can be compared statistically to other instruments. The WDQ was specifically designed to reflect different aspects of health status for patients with WAD, this is reflected in relatively low Pearson’s r with change in the other instruments from ME1 to ME3. Relations with external criteria are of some interest, but they do not not provide information on whether it actually assesses the construct in a more specific way. Once a German version of a closely related instrument is available that has been evaluated for psychometric properties, e.g. the Neck Disability Index, a comparison of both instruments’ responsiveness would be of interest.

Although the WDQ-G is not necessarily more precise than concurrent instruments, it is easier to apply and quicker to fill in than many of the available questionnaire’s, while at the same time being informative on all dimensions.

## Conclusion

WADs comprise a row of different symptoms that are difficult to track reliably for professional treatment. So far, there was no WAD-specific self-administered questionnaire covering all aspects of impairment (pain levels, personal care, role performance, mobility, sleep disturbances, tiredness, social and leisure (sporting and non-sporting) activity, emotional and cognitive impairments). With the English version of the WDQ a long overdue assessment for practitioners and researchers to monitor patients with WAD has been developed and reliability and responsiveness of its translation are presented here. The study showed that the WDQ-G is a reliable questionnaire with comparable responsiveness as traditional health related outcomes for whiplash. The decision as to how much change is relevant might have to be based on the individual case. The study provides necessary results for the clinician to decide, with which certainty a specific cut-off point on the WDQ-G can be deemed improved. A change of 11 points represents MIC from a patient’s perspective with highest sensitivity and specificity. Using the MDC with 17 points change as cut-off has lower sensitivity but is more accurate from a statistical point of view. Change in 15 points could be used as middle course, with 90% confidence for true change and high specificity (86%) but reduced sensitivity (64%).

The German version of the WDQ can be obtained free of charge from the second author: Dr. Corina Schuster: c.schuster@reha-rhf.ch.

## Abbreviations

CI: Confidence interval; HTI: Health transition item of the SF-36 (item 2); ICC: Intraclass correlation coefficient; MDC: Minimal detectable change; ME: Measurement event; MIC: Minimal important change; MTBI: Mild traumatic brain injury; N: Sample size; NASS: North American spine society questionnaire; NASS-PF: Pain & function subscale of NASS; QTF: Quebec task force; SEM: Standard error of measurement; SF-36: Medical outcomes study (MOS) 36-item short form health survey; SF-36BP: Bodily pain subscale of the SF-36; SPSS: Statistical package for social sciences; SRM: Standardised response mean; VAS: Visual analogue scale for pain; WAD: Whiplash associated disorders; WDQ: Whiplash disability questionnaire; WDQ-G: Whiplash disability questionnaire German version.

## Competing interests

The authors declare that they have no financial or non-financial competing of interests.

The preliminary results have been presented as a poster at the 5th Word Conference on Neuro Rehabilitation in Brasilia 2008 and at the 16th World Congress for Physical Therapy in Amsterdam 2011.

## Authors’ contributions

CS was the project leader. She wrote the study protocol, administered the questionnaires and collected the data. She helped with data interpretation and critically reviewed the manuscript. MM analysed and interpreted the data, and wrote the manuscript. TE was involved in study design, data interpretation and he critically revised the manuscript. All authors gave final approval of the manuscript.

## Funding

The study was partially funded by the Foundation for Research in Public Health of the Canton Aargau, Switzerland.
